# Iron Limitation Modulates Ocean Acidification Effects on Southern Ocean Phytoplankton Communities

**DOI:** 10.1371/journal.pone.0079890

**Published:** 2013-11-20

**Authors:** Clara J. M. Hoppe, Christel S. Hassler, Christopher D. Payne, Philippe D. Tortell, Björn Rost, Scarlett Trimborn

**Affiliations:** 1 Alfred Wegener Institute Helmholtz Centre for Polar and Marine Research, Bremerhaven, Germany; 2 University of Technology Sydney, Plant Functional Biology and Climate Chance Cluster, New South Wales, Australia; 3 University of British Columbia, Vancouver, British Columbia, Canada; The Evergreen State College, United States of America

## Abstract

The potential interactive effects of iron (Fe) limitation and Ocean Acidification in the Southern Ocean (SO) are largely unknown. Here we present results of a long-term incubation experiment investigating the combined effects of CO_2_ and Fe availability on natural phytoplankton assemblages from the Weddell Sea, Antarctica. Active Chl *a* fluorescence measurements revealed that we successfully cultured phytoplankton under both Fe-depleted and Fe-enriched conditions. Fe treatments had significant effects on photosynthetic efficiency (F_v_/F_m_; 0.3 for Fe-depleted and 0.5 for Fe-enriched conditions), non-photochemical quenching (NPQ), and relative electron transport rates (rETR). pCO_2_ treatments significantly affected NPQ and rETR, but had no effect on F_v_/F_m_. Under Fe limitation, increased pCO_2_ had no influence on C fixation whereas under Fe enrichment, primary production increased with increasing pCO_2_ levels. These CO_2_-dependent changes in productivity under Fe-enriched conditions were accompanied by a pronounced taxonomic shift from weakly to heavily silicified diatoms (i.e. from *Pseudo-nitzschia* sp. to *Fragilariopsis* sp.). Under Fe-depleted conditions, this functional shift was absent and thinly silicified species dominated all pCO_2_ treatments (*Pseudo-nitzschia* sp. and *Synedropsis* sp. for low and high pCO_2_, respectively). Our results suggest that Ocean Acidification could increase primary productivity and the abundance of heavily silicified, fast sinking diatoms in Fe-enriched areas, both potentially leading to a stimulation of the biological pump. Over much of the SO, however, Fe limitation could restrict this possible CO_2_ fertilization effect.

## Introduction

The Southern Ocean (SO) exerts a disproportionate control on the global carbon cycle over glacial-interglacial timescales [1], [2] and contributes significantly to the oceanic sequestration of anthropogenic CO_2_ [3]. Besides abiotic drivers such as ocean circulation and sea-ice cover, biological carbon uptake and drawdown also control the air-sea-flux of CO_2_ in the SO [1], [2]. These biological processes are mediated by phytoplankton communities, dominated mainly by silicifying diatoms [4].

The surface waters of the SO are rich in major nutrients such as nitrate and phosphate, but in vast areas of this region primary production is limited by low iron (Fe) availability [5]. Both laboratory and *in-situ* fertilisation experiments have demonstrated that the growth of SO phytoplankton is strongly enhanced by the addition of Fe [6], [7], [8]. As Fe is a key nutrient for biochemical pathways including photosynthesis and nitrate assimilation [9], limiting Fe concentrations lead to decreased photochemical efficiencies and photosynthetic rates [10], [11]. One important source of iron in open-ocean waters is the melting of sea-ice, which causes seasonal and localized phytoplankton blooms and strong vertical particle fluxes [12], [13]. These factors make the marginal sea-ice zone a biogeochemically important region of the SO [12].

The effects of seawater carbonate chemistry on SO phytoplankton have received increasing attention over recent years. Laboratory studies suggest that Antarctic phytoplankton can be growth-limited by CO_2_ supply under present-day CO_2_ concentrations [14], [15]. Field data from continental shelf waters of the Ross Sea have demonstrated CO_2_-dependent changes in primary productivity and phytoplankton assemblages [16], [17]. In these prior studies, phytoplankton assemblages were not demonstrably Fe-limited (e.g. high F_v_/F_m_ reported in [17]), making the extrapolation of results to the open SO waters difficult. Recently, pH-dependent shifts in Fe speciation have been reported [18], suggesting a strong potential for ocean acidification (OA) to reduce Fe bioavailability as seen in experiments with Arctic phytoplankton assemblages [19].

Given the Fe-limited status of much of the SO, there is a great need to investigate combined effects of OA and Fe limitation in this region. Here we present results from a CO_2_-Fe-incubation experiment (190, 390 and 800 μatm pCO_2_ under Fe-enriched and Fe-depleted conditions) using an open ocean phytoplankton assemblage from the Weddell Sea, an important region for SO primary productivity. The aim of this study was to investigate the interactive effects of OA and Fe availability on species composition, primary production, as well as iron uptake and photophysiology of Fe-limited phytoplankton assemblages.

## Materials and Methods

### Experimental Setup

A ship-board incubation experiment was designed using a CO_2_-Fe-matrix-approach to examine potential interactive effects between CO_2_ and Fe availability on SO phytoplankton communities. A natural phytoplankton assemblage from the Weddell Sea (66°50′S, 0°W) was sampled during mid Dec. 2010 on the *RV Polarstern* ANT-XXVII/2 cruise. The permission for field work according to the Antarctic Treaty was issued by the Umweltbundesamt (Germany). Seawater was collected from 30 m depth using a “torpedo fish” towed outside the ship's wake [20]. To eliminate large grazers, we filtered seawater through an acid-cleaned 200 µm mesh. Water containing the natural phytoplankton community was transferred into acid-cleaned 4L polycarbonate bottles and incubated in growth chambers at 3±1°C with a constant daylight irradiance of 40±5 µmol photons m^−2^ s^−1^ (Master TL-D 18W daylight lamps, Philips, adjusted by neutral density screens). The applied irradiance was based on several light measurements in the SO at the sampling depth (data by Mitchell; e.g. DOI: 10.1594/PANGAEA.132802). To provide sufficient time for changes in the phytoplankton assemblages to occur and achieve ecologically relevant information, experiments lasted between 18 and 30 days depending on experimental treatment (18–20 days in case of Fe-enriched and 27–30 days in case of Fe-depleted treatments). In order to prevent significant changes in chemical conditions due to phytoplankton growth, incubations were diluted with 0.2 µm filtered seawater when nitrate concentrations were about 10 µmol kg^−1^. Dilution water was obtained from the initial sampling location and filtered through acid-cleaned 0.2 µm filter cartridges (AcroPak 1500, PALL). Experiments were run with triplicate treatments of two Fe levels (Fe-enriched and Fe-depleted; see below) and 3 pCO_2_ levels (190, 390 and 800 μatm).

Tubing, bubbling systems, reservoir carboys, incubation bottles and other equipment were acid-cleaned prior to the cruise using trace metal-clean techniques: After a 2-day Citranox detergent bath and subsequent rinsing steps with Milli-Q (MQ, Millipore), equipment was kept in acid (5N HCl for polyethylene and 1N HCl for polycarbonate materials) for 7 days, followed by 7 rinses with MQ. Equipment was kept triple-bagged during storage and experiments. Incubation bottles were stored under acidified conditions (addition of 500 µL 10N suprapure quarz destilled HCl, Carl Roth, in 500 mL MQ) and rinsed twice with seawater prior to the start of the experiment.

In order to mimic different pCO_2_ conditions, the incubation bottles were continuously sparged with air of different CO_2_ partial pressures (190, 390 and 800 μatm) delivered through sterile 0.2 um air-filters (Midisart 2000, Sartorius stedim). Gas mixtures were generated using a gas flow controller (CGM 2000 MCZ Umwelttechnik), in which CO_2_-free air (<1 ppmv CO_2_; Dominick Hunter) was mixed with pure CO_2_ (Air Liquide Deutschland). The CO_2_ concentration in the mixed gas was regularly monitored with a non-dispersive infrared analyzer system (LI6252, LI-COR Biosciences) calibrated with CO_2_-free air and purchased gas mixtures of 150±10 and 1000±20 ppmv CO_2_ (Air Liquide Deutschland).

To promote phytoplankton growth, 1 nM Fe (FeCl_3_, ICP-MS standard, TraceCERT, Fluka) was added to the Fe-enriched treatments. In the Fe-depleted treatments, 10 nM of the hydroxamate siderophore desferrioxamine B (DFB, Sigma) was added to bind and thereby reduce the bioavailable Fe [21], [22]. No additional macronutrients were added to the incubation bottles. Abiotic control bottles, used to assess changes in Fe chemistry, contained filtered seawater (0.2 µm) exposed to each treatment condition over the duration of the experiment.

### Chemical parameters

Nutrients were determined colorimetrically on-board with a Technicon TRAACS 800 Auto-analyzer on a daily basis over the course of the experiments, following procedures improved after [23]. Samples for total alkalinity (TA) were 0.6 µm-filtered (glass fibre filters, GF/F, Whatman), fixed with 0.03% HgCl_2_ and stored in 150 mL borosilicate bottles at 4°C. TA was estimated at the Alfred Wegener Institute (Germany) from duplicate potentiometric titration [24] using a TitroLine alpha plus (Schott Instruments). The calculated TA values were corrected for systematic errors based on measurements of certified reference materials (CRMs provided by Prof. A. Dickson, Scripps, USA; batch #111; reproducibility ±5 µmol kg^−1^). Dissolved inorganic carbon (DIC) samples were filtered through 0.2 µm cellulose-acetate filters (Sartorius stedim), fixed with 0.03% HgCl_2_ and stored in 5 mL gas-tight borosilicate bottles at 4°C. Also in the home laboratory, DIC was measured colourimetrically in triplicate with a QuAAtro autoanalyzer (Seal) [25]. The analyser was calibrated with NaHCO_3_ solutions (with a salinity of 35, achieved by addition of NaCl) with concentrations ranging from 1800 to 2300 µmol DIC kg^−1^. CRMs were used for corrections of errors in instrument performance (e.g. baseline drift). Seawater pH was measured potentiometrically on the NBS scale (pH_NBS_; overall uncertainty 0.02 units) with a two-point calibrated glass reference electrode (IOline, Schott Instruments). Values for pH were reported on the pH_total_ scale for better comparability with other datasets. Following suggestions by Hoppe et al. [26], seawater carbonate chemistry (including pCO_2_) was calculated based on TA and pH using CO_2_SYS [27]. The dissociation constants of carbonic acid of Mehrbach et al. (refit by Dickson and Millero) were used for calculations [28], [29]. Dissociation constants for HSO_4_ were taken from Dickson [30]. Iron chemistry was analyzed using the competitive ligand exchange adsorptive cathodic stripping voltammetry using the ligand 2-(2-thiazolylazo)-p-cresol (TAC, 10 µmol kg^−1^ [31]). Total dissolved (<0.2 um) Fe concentrations were analyzed following a 45 min UV-photo-oxidation step (acid washed quartz tubes closed with Teflon lids) and concentrations were determined in triplicate using standard additions of a freshly made FeCl_3_ standard (ICP-MS standard, TraceCERT, Fluka, 1–4 nM).

### Biological Parameters

To determine the taxonomic compositions at the end of the experiment, duplicate aliquots of 200 mL unfiltered seawater were preserved with both hexamine-buffered formalin solution (2% final concentration) and Lugols (1% final concentration). Preserved samples were stored at 4°C in the dark until further analysis by inverted light microscopy (Axiovert 200, Zeiss). Additionally, species dominating the final communities were identified using scanning electron microscopy (Philips XL30) according to taxonomic literature [32]. Average biovolume of the dominant species was calculated based on representative cell size measurements [33]. Values were in good agreement with the MAREDAT database [34]. For analysis of particulate organic carbon (POC), cells were collected onto precombusted GF/F-filters (15 h, 500°C), which were subsequently stored at −20°C and dried for >12 h at 60°C prior to sample analysis. Analysis was performed using an Automated Nitrogen Carbon Analyser mass spectrometer system (ANCA-SL 20-20, SerCon Ltd.). Samples for determination of chlorophyll *a* (Chl *a*) concentration were filtered onto 0.45 µm cellulose acetate filters (Sartorius stedim) and stored at −20°C until analysis onboard. Chl *a* was subsequently extracted in 10 mL 90% acetone (overnight in darkness, at 4°C) and concentrations determined on a fluorometer (10-000 R, Turner Designs), using an acidification step to determine phaeopigments [35].

### Physiological assays

Primary production of the final phytoplankton assemblages was determined in 100 mL incubations after addition of a 10 µCi (0.37 MBq) spike of NaH^14^CO_3_ (PerkinElmer, 53.1 mCi mmol^−1^). From the incubations, 0.5 mL aliquots were immediately removed and mixed with 10 mL of scintillation cocktail (Ultima Gold AB, PerkinElmer) to determine the total amount of added NaH^14^CO_3_. For blank determination, samples were filtered and acidified immediately after ^14^C spikes. After 24 h of incubation under acclimation light intensity, samples were filtered onto GF/F-filters, acidified with 6N HCl and left to degas overnight. Filters were then transferred into scintillation vials, to which 10 mL of scintillation cocktail was added. After ≥2 h, the samples were measured on a ship-board liquid scintillation counter (Tri-Carb 2900TR, PerkinElmer), using automatic quench correction and a maximum counting time of 5 minutes.

Maximum Fe uptake capacity of the final phytoplankton assemblages was estimated after 2–4 h dark-acclimation in 500 mL acid-cleaned PC-bottles. In case of the Fe- treatments, cells were gently concentrated by filtration over an HCl-cleaned 2 µm membrane filter (Isopore, Millipore), rinsed and resuspended in 500 mL filtered seawater from the initial sampling location in order to dispose all DFB. From this, 50 mL were taken for Chl *a* measurements. Subsequently, 1 nM of ^55^Fe (Perkin Elmer, 33.84 mCi mg^−1^ as FeCl_3_ in 0.5 N HCl) was added to each sample (both Fe- and -enriched). Generally, 2 mL were taken from all samples to determine the initial amount of ^55^Fe. Subsequently, cells were exposed for at least 24 h to the acclimation light intensity. At the end of the incubation time, the sample was filtered onto GF/F-filters and rinsed 5 times with oxalate solution (gravity filtered, 2 min between rinses) and 3 times with natural seawater [36]. Each filter was then collected in a scintillation vial, amended with 10 mL scintillation cocktail (Ultima Gold A, PerkinElmer) and mixed thoroughly (Vortex). ^55^Fe counts per minute were estimated for each sample on the ship-board liquid scintillation counter (Tri-Carb 2900TR, PerkinElmer), and converted into disintegrations per minute considering the radioactive decay and custom quench curves. ^55^Fe uptake was then calculated taking into account the initial ^55^Fe concentration and the total dissolved Fe concentration (background and added). Fe uptake rates were normalized to POC using Chl *a*∶POC ratios from the respective treatments.

Photophysiological parameters were measured using a Fluorescence Induction Relaxation System (FIRe; Satlantic, Canada; 37). Samples were 1 h dark-acclimated prior to measurements to ensure that all photosystem II (PSII) reaction centers were fully oxidized and non-photochemical quenching (NPQ) was relaxed. The duration of the dark acclimation was chosen after testing different time intervals (data not shown). Samples were then exposed to a strong short pulse (Single Turnover Flash, STF), which was applied in order to cumulatively saturate PSII. Afterwards, a long saturating pulse (Multiple Turnover Flash, MTF) was applied in order to fully reduce the PSII and the plastoquinone (PQ) pool. The minimum (F_0_) of the STF phase and maximum (F_m_) fluorescence of the MTF was used to calculate the apparent maximum quantum yield of photochemistry in PSII (F_v_/F_m_) according to the equation (F_m_−F_0_)/F_m_. This parameter was calculated for all bottles on a regular basis (every 6–7 days). Values of these parameters as well as of the functional absorption cross section of PSII (σ_PSII_ [Å^2^ quanta^−1^]) were derived using the FIRePro software provided by Satlantic [37]. Additional fluorescence measurements were performed under increasing irradiances (21, 41, 66, 88, 110 and 220 µmol photons m^−2^ s^−1^) provided by an external actinic light source (warm white 350 mA LEDs, ILL3A003, CML Innovative Technologies). After 5 minutes acclimation to the respective light level, the light-acclimated minimum (F_q_′) and maximum (F_m_′) fluorescence were estimated. The effective quantum yield of photochemistry in open reaction centers of PSII was derived according to the equation (F_m_′−F_q_′)/F_m_′ [38]. Relative electron transport rates (rETR) were then calculated as the product of effective quantum yield and applied growth irradiance of 40 µmol photons m^−2^ s^−1^. Using the Stern-Volmer equation [39], NPQ of Chl *a* fluorescence under growth irradiance was calculated as F_m_/F_q_′−1. NPQ was relaxed (values <0.1) at lowest light levels for all treatments (data not shown). All measurements were conducted at the growth temperature.

### Statistics

All data is given as the mean of the replicates (*n* = 3) with 1 standard deviation. To test for significant differences between the treatments, Two Way Analyses of Variance (ANOVA) with additional normality tests (Shapiro-Wilk; passed for all data shown) were performed. The significance level was set to 0.05. Statistical analyses were performed with the program SigmaPlot (SysStat Software Inc).

## Results

### Seawater chemistry

**Figure 1:**
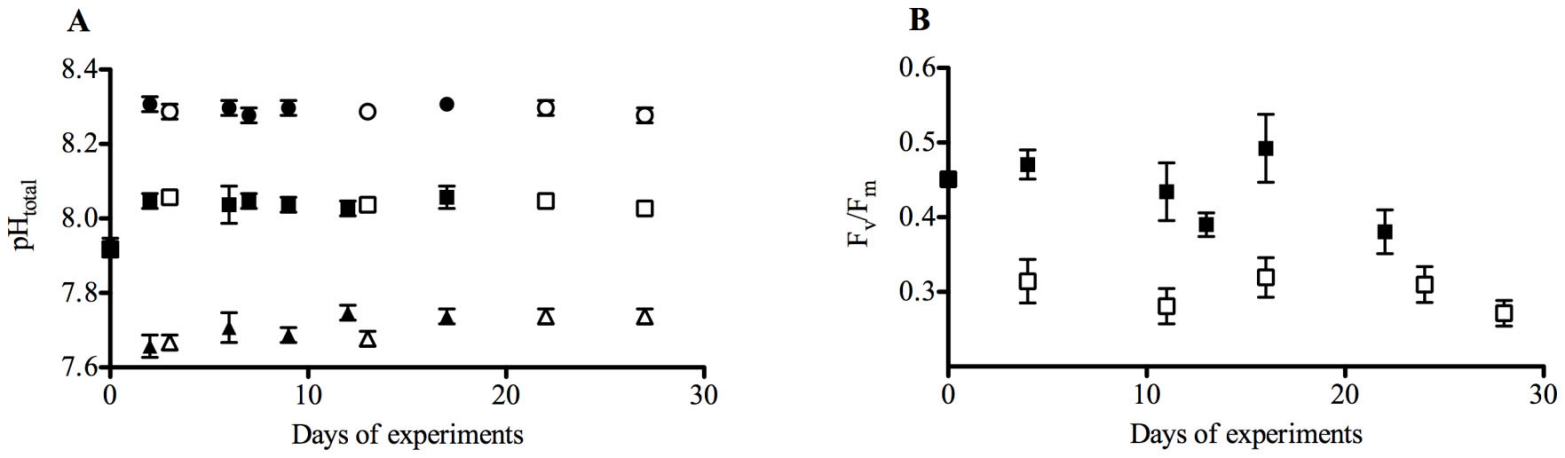
pH and F_v_/F_m_ over the course of the experiment. Experimental conditions over the course of the experiment. A: Development of pH_total_ (n = 3; mean ±1 s.d.) in Fe-enriched (solid circles, 190 μatm CO_2_; solid squares, 390 μatm CO_2_; solid triangles, 800 μatm CO_2_) and Fe-depleted treatments (open circles, 190 μatm CO_2_; open squares, 390 μatm CO_2_; open triangles, 800 μatm CO_2_). B: Development of dark-adapted F_v_/F_m_ (n = 3; mean ±1 s.d.) in Fe-enriched (solid squares) and Fe-depleted treatments (open squares).

The initial carbonate system (pH: 7.93±0.01; DIC 2210±17 µmol kg^−1^; TA: 2303±14 µmol kg^−1^) shifted to experimental treatment levels (average pH of 8.39±0.02, 8.13±0.02, and 7.80±0.03 for the three CO_2_ treatments) within the first 2 days of the experiment (Figure 1 A). The semi-continuous dilute-batch approach led to stable seawater carbonate chemistry over the course of the experiment (Figure 1 A). Compared to abiotic controls, drift was <8% and <5% for TA and DIC, respectively (Table 1). Initial seawater nutrient concentrations were 29 µmol kg^−1^ nitrate, 76 µmol kg^−1^ silicate and 2 µmol kg^−1^ phosphate. Over the course of the experiment, concentrations of nitrate never fell below 7 µmol kg^−1^, while silicate and phosphate concentrations were always above 40 and 0.8 µmol kg^−1^, respectively. Initial Fe concentration in the water sampled for incubations was 1.12±0.15 nmol kg^−1^. In 0.2 µm filtered seawater (i.e. abiotic control treatments) enriched with 10 nM DFB, dissolved Fe concentrations remained 1.16±0.08 nmol kg^−1^ until the end of the experiment (Table 1), indicating that the experimental bottles remained free of Fe contamination. Dissolved Fe concentrations decreased in Fe-enriched seawater (1 nM Fe added, Table 1).

**Table 1:** Seawater chemistry.

Treatment		Fe_diss_		DIC		TA		pH		pCO_2_	
		[µmol kg^−1^]		[µmol kg^−1^]		[µmol kg^−1^]		[total]		[μatm]	
Initial seawater	1.12	±0.15	2071		2271		7.93		518		
+Fe	190 μatm CO_2_	0.45	±0.07	2002	±11	2208	±36	8.31	±0.01	188	±6
	390 μatm CO_2_	0.32	±0.04	2082	±4	2230	±8	8.06	±0.03	369	±30
	800 μatm CO_2_	0.21	±0.03	2175	±14	2215	±8	7.74	±0.02	801	±51
−Fe	190 μatm CO_2_	1.13	±0.16	2018	±18	2209	±5	8.28	±0.01	204	±5
	390 μatm CO_2_	1.25	±0.21	2096	±41	2219	±21	8.03	±0.02	398	±22
	800 μatm CO_2_	1.11	±0.09	2155	±41	2241	±52	7.74	±0.01	813	±11

Parameters of the seawater carbonate chemistry were sampled at the beginning (*n* = 1) and the end of the experiment (*n* = 3; mean ±1 s.d.). Total dissolved Fe measurements in abiotic control treatments after 0.2 µm filtration as measured by voltammetry (*n* = 2; mean ± 1 s.d.). The decreased dissolved Fe concentration in the +Fe treatment can be attributed to precipitation/absorption of colloidal iron. pCO_2_ was calculated from TA and pH_total_ at 3°C and a salinity of 34 using CO_2_SYS [27], using average final nutrient concentrations of 1 and 60 µmol kg^−1^ for phosphate and silicate, respectively.

### Photophysiology

Over the course of the experiment, we observed significant Fe-dependent differences in the apparent maximum quantum yield of PSII reaction centres (F_v_/F_m_; Figure 1 B; p<0.001). Average values of F_v_/F_m_ at the end of the experiment were 0.51±0.04 and 0.32±0.03 for Fe-enriched and -depleted treatments, respectively (Table 2). In contrast, the pCO_2_ treatments had no effects on F_v_/F_m_ under either of the Fe treatments. Relative electron transfer rates from PSII (rETR) were significantly higher in Fe-enriched treatments, and also showed pCO_2_-dependent increases under both Fe-enriched (5.7±0.8 at low and 7.5±0.9 at high pCO_2_; p<0.001) and Fe-depleted conditions (4.2±0.4 at low and 5.3±0.1 at high pCO_2_; p<0.001). Significant interactive effects between Fe- and pCO_2_-treatments were also observed in photoprotective non-photochemical quenching (NPQ; Table 2; p<0.001). Under Fe-limitation, NPQ decreased from 0.22±0.03 at low pCO_2_ to 0.11±0.00 at high pCO_2_, while under Fe-enriched conditions, NPQ was independent of pCO_2_ (0.06±0.01 at all pCO_2_ levels; p<0.001).

**Table 2:** Physiological differences between treatments.

Treatment		Chl a∶ POC			F_v_/F_m_			σ_PSII_			NPQ			rETR		
+Fe	190 μatm CO_2_	0.023	±0.003	F_[Fe]_ = 59.57	0.55	±0.02	F_[Fe]_ = 162.69	270	±37.1	F_[Fe]_ = 36.89	0.06	±0.00	F_[Fe]_ = 166.34	5.72	±0.78	F_[Fe]_ = 59.03
	390 μatm CO_2_	0.025	±0.005	**p_[Fe]_<0.001**	0.50	±0.03	**p_[Fe]_<0.001**	230	±34.9	**p_[Fe]_<0.001**	0.05	±0.02	**p_[Fe]_<0.001**	5.47	±1.32	**p_[Fe]_<0.001**
	800 μatm CO_2_	0.017	±0.004	F_CO2_ = 6.84	0.52	±0.01	F_CO2_ = 0.02	229	±19.5	F_CO2_ = 5.29	0.06	±0.01	F_CO2_ = 0.34	7.5	±0.87	F_CO2_ = 22.75
−Fe	190 μatm CO_2_	0.012	±0.001	**p_CO2_ = 0.012**	0.31	±0.02	p_CO2_ = 0.894	345	±21.1	p_CO2_ = 0.136	0.22	±0.03	p_CO2_ = 0.568	4.16	±0.40	**p_CO2_<0.001**
	390 μatm CO_2_	0.013	±0.001		0.33	±0.03		330	±26.1		0.19	±0.02	F_[Fe],CO2_ = 36.07	4.48	±0.34	
	800 μatm CO_2_	0.01	±0.001		0.32	±0.02		301	±35.9		0.11	±0.00	**p_[Fe],CO2_<0.001**	5.29	±0.08	

Final Chl a∶ POC ratios (µg∶µg) and photophysiological parameters (apparent maximum quantum yield of PSII F_v_/F_m_, proportion of non-photochemical quenching, NPQ, functional absorption cross section of PSII (σ_PSII_ [Å^2^ quanta^−1^]), and relative electron transport rates from PSII, rETR) of the final communities grown under different CO_2_ and Fe levels (n = 3; mean ±1 s.d.). Fe-depleted (−Fe) and Fe-enriched (+Fe) conditions were achieved by the addition of 10 nmol kg^−1^ DFB and 1 nmol kg^−1^ FeCl_3_, respectively. Bold *p* values indicate statistically significant differences between treatments (p<0.05; 2-way ANOVA).

The ratios of Chl *a* to POC of the final phytoplankton assemblages (Table 1) were significantly higher (p<0.001) in Fe-enriched (0.023±0.003 at low and 0.017±0.004 at high pCO_2_) compared to Fe-depleted treatments (0.012±0.001 at low and 0.010±0.001 at high pCO_2_). Chl *a*∶POC ratios furthermore decreased significantly with increasing pCO_2_ levels (p = 0.012), irrespective of the Fe-status.

### Fe and C uptake

**Figure 2:**
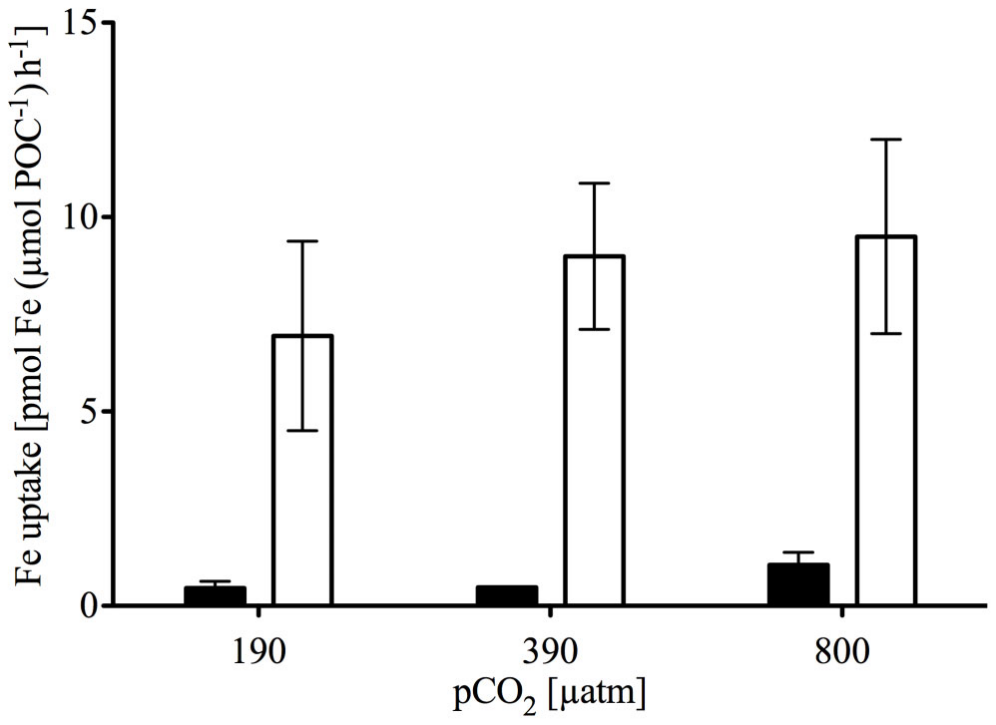
Iron uptake capacities of final phytoplankton communities. Fe uptake capacities [pmol Fe (µmol POC)^−1^ h^−1^] of Fe-enriched (solid bars) and -depleted (open bars) final phytoplankton communities (n = 3; mean ±1 s.d.) estimated from 24 h incubation with 1 nM ^55^Fe as a function of pCO_2_ [μatm]. Statistical analysis (2-way ANOVA) revealed significant differences between Fe-treatments (F = 62.217, p = <0.001) but not between CO_2_-treatments (F = 1.205, p = 0.349).

**Figure 3:**
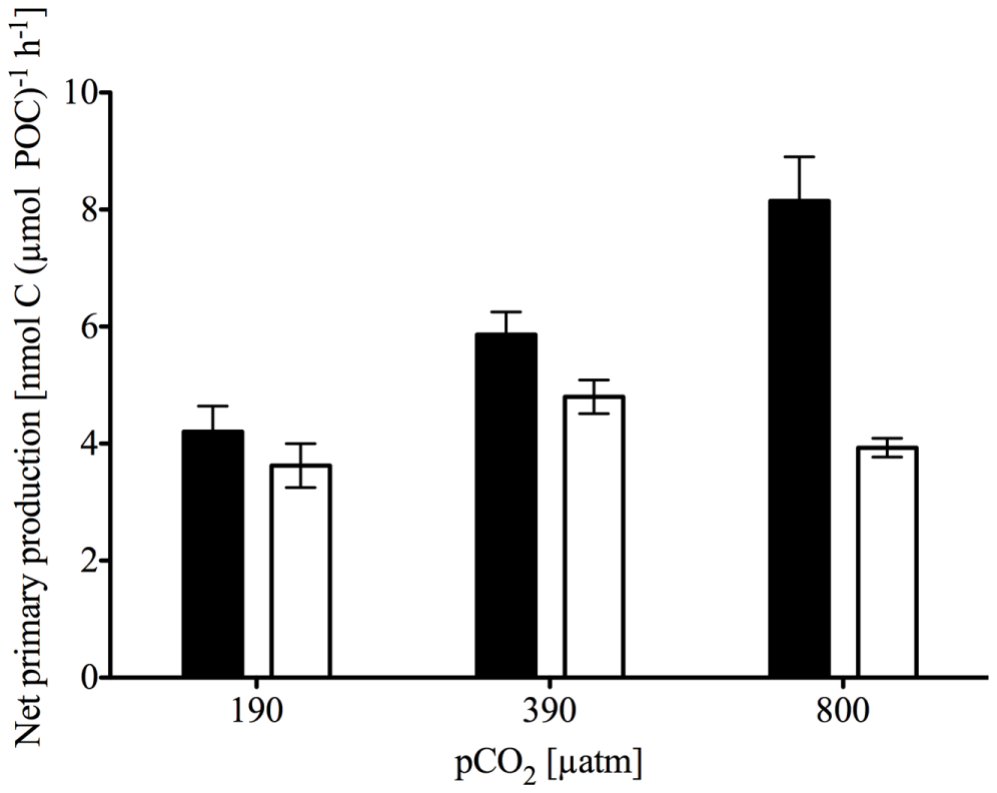
Net primary production of final phytoplankton communities. NPP [nmol C (µmol POC)^−1^ h^−1^] was estimated from ^14^C incubations over 24 h as a function of pCO_2_ [μatm]. Black and grey bars indicate Fe-enriched (solid bars) and -depleted treatments (open bars), respectively (n = 3; mean ± s.d.). ANOVA analysis revealed significant effects of pCO_2_ levels as well as a significant interaction term of [Fe] and pCO_2_ levels (F_[Fe]_ = 0.01, p_[Fe]_ = 0.92; F_pCO2_ = 15.56, p_pCO2_<0.001; F_[Fe],pCO2_ = 5.85, p_[Fe],pCO2_ = 0.023).

For all pCO_2_ levels, carbon-normalized Fe uptake capacities at the end of the experiment were 10-fold higher in Fe-depleted compared to Fe-enriched treatments (Figure 2; p<0.001), but with no significant CO_2_ effect. Combining data across pCO_2_ treatments, mean Fe uptake capacities were 7.50±3.35 pmol Fe (µmol POC)^−1^ h^−1^ and 0.72±0.38 pmol Fe (µmol POC)^−1^ h^−1^ at low and high Fe, respectively. Under Fe-enriched conditions, we observed a significant CO_2_-dependent increase in C-specific primary productivity (Figure 3; p<0.001). Primary productivity increased from 4.21±0.44 nmol C (µmol POC)^−1^ h^−1^ at low pCO_2_ to 8.15±0.75 nmol C (µmol POC)^−1^ h^−1^ at high pCO_2_. In contrast, no CO_2_-dependent productivity responses were observed in Fe-depleted treatments, with values of 3.62±0.38 nmol C (µmol POC)^−1^ h^−1^ at low pCO_2_ and 3.93±0.16 nmol C (µmol POC)^−1^ h^−1^ at high pCO_2_. Thus, there was a significant interactive effect of the CO_2_- and Fe-treatments on NPP (p = 0.023).

### Species composition

**Figure 4:**
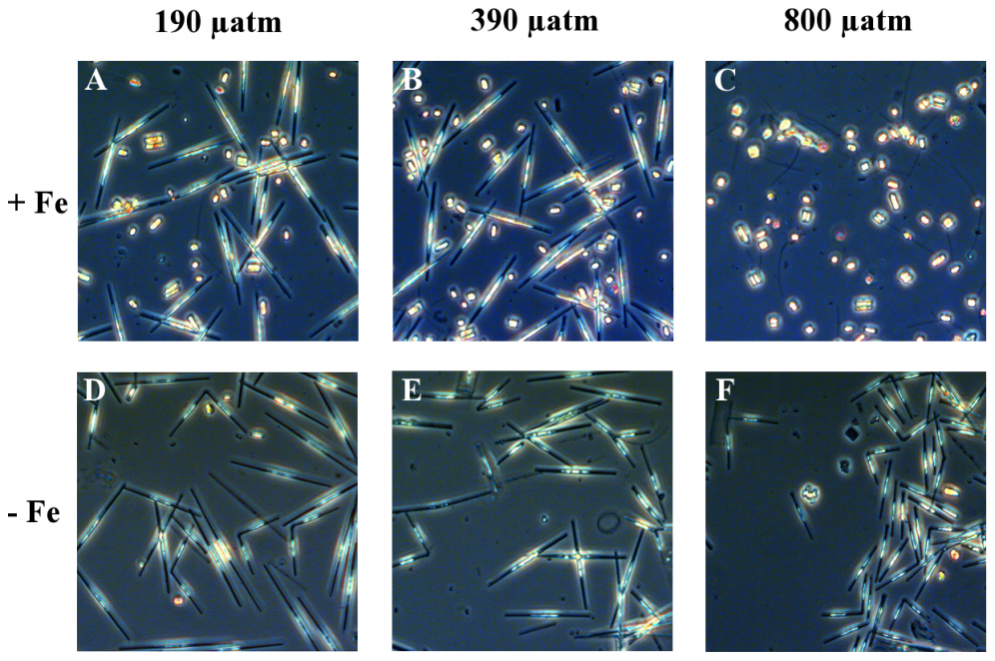
Representative microscopy pictures of species composition of the final communities. A, Fe-enriched 190 μatm (39±5% *Pseudo-nitzschia*, 43±4% *Fragilariopsis*); B, Fe-enriched 390 μatm (40±9% *Pseudo-nitzschia*, 42±12% *Fragilariopsis*); C, Fe-enriched 800 μatm (72±5% *Fragilariopsis*); D, Fe-depleted 190 μatm (55±16% *Pseudo-nitzschia*, 26±20% *Synedropsis*); E, Fe-depleted 390 μatm (51±15% *Pseudo-nitzschia*, 29±16% *Synedropsis*); F, Fe-depleted 800 μatm (78±2% *Synedropsis*).

We observed pronounced shifts in the diatom-dominated phytoplankton assemblages in association with the CO_2_-dependent changes in primary productivity (Figure 4; Table 3). Shifts in species composition did not lead to changes in average cell size in the different assemblages (data not shown). After Fe-enrichment, *Pseudo-nitzschia* cf. *turgiduloides* was the most abundant species under low and intermediate pCO_2_ (39±5% and 40±9%, respectively)_,_ whereas *Fragilariopsis cylindrus* dominated communities under high pCO_2_ levels (72±5%). Furthermore, *Chaetoceros* cf. *simplex* abundances increased with rising CO_2_ (from 11±0% at low pCO_2_ to 17±1% at high pCO_2_). Phytoplankton composition changes were also observed under Fe limitation, but the nature of these species shifts differed significantly from those seen under high Fe levels (Figure 4; Table 3). Under Fe-depleted conditions, *Pseudo-nitzschia* cf. *turgiduloides* dominated the low pCO_2_ treatment (55±16%), while *Synedropsis* sp. was the most prevalent species under high pCO_2_ (78±2%).

**Table 3:** Microscopic cell counts.

Taxonomic group	Initial	+Fe						−Fe					
		190 μatm		390 μatm		800 μatm		190 μatm		390 μatm		800 μatm	
*Pseudo-nitzschia* cf. *turgiduloides*	5	39	±5	40	±9	3	±3	55	±16	51	±15	<0.5	
*Synedropsis* sp.	1	1	±1	1	±0	3	±2	26	±20	29	±16	78	±2
*Fragilariopsis cylindrus*	32	43	±4	42	±12	72	±5	3	±2	2	±1	5	±3
*Chaetoceros* cf. *simplex*	6	11	±0	10	±2	17	±1	1	±1	3	±2	1	±1
*Phaeocystis antarctica*	18	1	±0	2	±2	2	±1	7	±2	6	±3	5	±1
unidentified flagellates	9	3	±1	2	±2	3	±3	<0.5		<0.5		3	±1
Choanoflagellates	6	<0.5		1	±1	<0.5		4	±2	6	±3	6	±0
*Ceratoneis closterium*	1	<0.5		<0.5		<0.5		1	±0	1	±1	1	±0
Dinoflagellates	6	<0.5		<0.5		<0.5		1	±1	<0.5		<0.5	
*Pseudo-nitzschia* cf. *turgidula*	2	<0.5		<0.5		<0.5		<0.5		<0.5		<0.5	
*Fragilariopsis kerguelensis*	7	<0.5		<0.5		<0.5		<0.5		<0.5		<0.5	
*Thalassiosira* sp.	3	<0.5		<0.5		<0.5		<0.5		<0.5		<0.5	
Large *Chaetoceros* sp.	2	<0.5		<0.5		<0.5		<0.5		<0.5		<0.5	
*Rhizosolenia* sp.	11	-		<0.5		<0.5		<0.5		<0.5		<0.5	
*Thalassiothrix* sp.	1	-		-		-		<0.5		-		-	
*Guinardia* sp.	<0.5	-		<0.5		<0.5		-		<0.5		<0.5	
*Eucampia* sp.	<0.5	<0.5		-		<0.5		-		<0.5		-	
Ciliates	<0.5	-		-		-		<0.5		<0.5		<0.5	
Silicoflagellates	<0.5	-		-		-		-		<0.5		-	

Species composition of the initial community (n = 1) and at the end of the experiment (% of total cell count; n = 3; average ±1s.d.).

## Discussion

### Confirmation of Fe limitation

Dissolved Fe concentrations in Fe-depleted abiotic controls remained at initial concentrations, showing that experimental manipulations and CO_2_ bubbling resulted in no significant Fe contamination (Table 1). In Fe-enriched abiotic controls, dissolved Fe concentrations decreased over the course of the experiment. This can be attributed to precipitation and absorption of colloidal iron in the absence of significant concentrations of Fe-binding ligands [40]. Fe limitation of phytoplankton in the DFB treatments was confirmed by significant differences in F_v_/F_m_ between Fe-enriched and -depleted treatments (Table 2; Figure 1 B). F_v_/F_m_ values of Fe-depleted treatments are comparable to those observed in naturally Fe-limited phytoplankton communities [41]. In line with previous findings on SO phytoplankton, also other photophysiological parameters like NPQ and rETR differed significantly between Fe treatments (Table 2, [42], [43]). Moreover, the significantly higher Fe uptake capacity in Fe-depleted treatments likely reflects the induction of high-affinity uptake systems and/or the selection of phytoplankton communities with greater Fe affinities [21], [22]. All of these observations confirm Fe limitation in the Fe-depleted treatments.

### OA response under Fe-enriched conditions

The observed CO_2_-dependent increase in primary productivity under Fe-enriched conditions (Figure 3) confirms that CO_2_ fixation in SO phytoplankton can be limited by carbon supply under current CO_2_ concentrations [14], [16]. This hypothesis is further supported by the decrease in Chl *a*∶POC ratios and the increase in rETRs with increasing pCO_2_ (Table 2). Similarly, Ihnken et al. [44] observed ETR_max_ in Fe-sufficient *Chaetoceros muelleri* to increase with increasing CO_2_. These findings suggest that the Calvin cycle, the major sink of photosynthetic energy [9], is the rate-limiting step of photosynthesis under low pCO_2_ levels. NPQ was not affected under high pCO_2_, Fe-enriched conditions. Under the applied irradiance of 40 µmol photons m^−2^ s^−1^, however, NPQ values were generally very low (Table 2) suggesting that there is little requirement for dissipation of light. For one of the dominant species in the Fe-enriched treatments, *Fragilariopsis cylindrus*, a significant induction of NPQ was only found at irradiances larger than 200 µmol photons m^−2^ s^−1^ [43]. We can therefore conclude that even when photosynthesis was carbon limited under low pCO_2_, the applied irradiance was too low to induce NPQ under Fe-enriched conditions and other pathways were operated as electron sinks (e.g. midstream-oxidases [9]).

The changes in physiological responses in Fe-enriched phytoplankton assemblages were accompanied by a pronounced shift in the species composition (Figure 4; Table 3) from *Pseudo-nitzschia* cf. *turgiduloides* under low and intermediate pCO_2_ to *Fragilariopsis cylindrus* under high pCO_2_ levels. Likely mechanisms for this floristic shift include species-specific differences in carbon acquisition [15], [45], as well as pH-mediated differences in cellular physiology, e.g. changes in electrochemical membrane potentials and ion transport processes [46]. *Pseudo-nitzschia* has also been observed to dominate in bloom situations after Fe fertilization [7], where pH increases due to biomass build-up and drawdown of CO_2_. This is in line with results from CO_2_ manipulations on SO phytoplankton assemblages, which were dominated by *Pseudo-nitzschia* at high pH [16]. In a laboratory study under Fe-enriched conditions, growth and rETRs of *Pseudo-nitzschia subcurvata* were unaffected by pCO_2_, suggesting that this species shows little to no responses to CO_2_ fertilization [15]. Our field experiment suggests that *Fragilariopsis cylindrus*, in contrast, benefited from increased *p*CO_2_. Even though information of OA responses for this species is lacking, the related *Fragilariopsis kerguelensis* showed enhanced rETRs with increasing *p*CO_2_ (S. Trimborn, unpublished data). We thus speculate that *F. cylindrus* increased its photosynthetic activity under elevated pCO_2_, thereby outcompeting the otherwise faster growing *Pseudo-nitzschia*. Under OA, also the relative abundance of *Chaetoceros* sp. increased by 50% (Table 3), which is consistent with previous findings on SO phytoplankton assemblages [16] as well as growth responses of *Chaetoceros debilis* to increased pCO_2_ [15].

### OA response under Fe-limitation

The observation that under Fe-limitation, productivity was not stimulated by increasing pCO_2_ (Figure 3) may indicate that Fe acts as the main limiting factor suppressing the effects of other nutrients such as inorganic carbon. Alternatively, the apparent insensitivity of primary production to OA may arise from antagonistic physiological responses to pCO_2_ and pH.

Under Fe limitation, elevated pCO_2_ significantly increased rETRs and decreased NPQ, while the functional absorption cross section was not affected (Table 2). These results suggest a greater electron sink associated with the Calvin cycle and thus a decreased need for energy dissipation under OA [44]. It is also known that linear electron transport (LET) towards the Calvin cycle is not the sole sink for photosynthetic energy and that, depending on the ATP demand of the cells, alternative electron pathways can play an important role (*e.g.* Mehler reaction, MOX pathway, pseudocyclic electron flow around PSI) [9]. Increased pCO_2_, however, is known to decrease photorespiration and/or the need for carbon concentrating mechanism (CCMs), which would lead to a decrease in cellular ATP demand [47]. The observed increase in rETRs in Fe-limited cells at high pCO_2_ levels (Table 2) may therefore rather be linked to higher LET rates than to alternative electron pathways. The higher LET, enabled by the enhanced CO_2_ fixation in the Calvin cycle, could counteract the generally greater need for photoprotection under Fe limitation [42], [43]. This could explain the opposing CO_2_ effects on NPQ under Fe-depleted and Fe-enriched conditions. The CO_2_ effect, apparent in photophysiology, is potentially masked in primary production by co-occurring pH effects on Fe bioavailability. According to the observed decline in Fe bioavailability with decreasing pH [18], [19], Fe-depleted phytoplankton would experience the greatest Fe stress under high pCO_2_.

In this study, Fe-limitation was achieved by the addition of the chelator DFB. Even though DFB has been shown to form strong complexes with Fe and thereby decrease Fe availability by >90% [36], phytoplankton can still access DFB-bound Fe to some extent [48], [49]. Since the phytoplankton assemblages in our DFB-treatments were strongly Fe-limited (as demonstrated by photophysiology, Fe and C uptake), bioavailability of Fe must have been largely reduced. Fe bioavailability also seems to be slightly reduced with increasing pCO_2_ (Figure 2), as has been observed in natural phytoplankton communities (i.e. without any added chelators) and in studies using different chelators such as EDTA and DFB [18], [19], [50]. Also, Maldonado et al. [49] suggest that the in-situ organic Fe-complexes observed in the SO have similar bioavailability compared to DFB. Although the chemical nature of in-situ organic Fe-binding ligands is not fully resolved, hydroxamate siderophores have been reported [51]. It is thus possible that at least some of the organically bound Fe exhibits a similar pH-dependent bioavailability as induced by DFB, and thus may allow for the extrapolation of our results to field situations. In order to study the bioavailability of Fe associated with in-situ Fe-binding organic ligands under OA scenarios, future experiments without added chelators should be conducted. As the nature of Fe-binding ligands remains largely unknown and can vary spatially [52], [53], one should address the Fe bioavailability of various compounds (e.g., humic acids, saccharids, exopolymeric substances) that were reported to affect iron biogeochemistry [36], [54], [55]. Furthermore, organic ligands control the bioavailability and the physico-chemistry of trace metals in general [52], [56]. As some of those (Co, Cd, Zn) are also essential for phytoplankton physiology (e.g. for the activity of the carbonic anhydrase) [57], joint measurements of other trace metals as well as their ligands would be desirable.

Although primary productivity was not sensitive to OA under Fe limitation, we did observe CO_2_-dependent species shifts, with *Pseudo-nitzschia* sp. dominating under low and *Synedropsis* sp. under high pCO_2_ (Figure 4). The low abundances of *F. cylindrus* in Fe-depleted treatments probably reflect the rather high sensitivity of this species towards Fe limitation [43], which could be due to low Fe uptake capacities observed for this species [21]. In contrast, *Pseudo-nitzschia* has been shown to be an efficient user of Fe under limiting concentrations [58] and sporadic Fe input events [59]. Interestingly, the final proportion of *Pseudo-nitzschia* declined strongly with increasing pCO_2_, irrespectively of the Fe status (Table 3), suggesting that its growth rates must have been significantly lower than those of the dominant species (*F. cylindrus* and *Synedropsis* sp. under Fe-enriched and -depleted conditions, respectively). This observation is in line with a recent study on *P. pseudodelicatissima*, whose growth rates were not affected by OA under either Fe-deplete nor -replete conditions [50]. As *Pseudo-nitzschia* generally does not seem to benefit from increased pCO_2_ levels ([15], [16], [50], this study), one could expect OA to have a negative effect on the abundances of this genus under on-going climate change. At present, nothing is known about the Fe and CO_2_ requirements of *Synedropsis* [60]. However, a possible appearance of *Synedropsis* in phytoplankton assemblages or incubation experiments might have been overlooked in past studies, as their delicately silicified frustules are very prone to dissolution [61] and not distinguishable from *Pseudo-nitzschia* by light microscopy (Figure 4).

Our results clearly demonstrate a strong difference in CO_2_-dependent community structure between Fe-enriched and Fe-depleted conditions (Figure 4). To explain these shifts, more information on species-specific differences in Fe requirements, uptake, as well as allocation strategies [21], [62], [63], and inorganic carbon acquisition [15], [16] is needed for all dominant species.

### Biogeochemical implications

The findings of our study suggest that the effects of OA on primary production and community structure are strongly modulated by the prevailing Fe concentrations. Our results, and those of others [16], [64], indicate a potential stimulation of the biological pump as a result of increased pCO_2_ in Fe-replete regions. Under Fe enrichment and increasing pCO_2_ levels, we observed a shift from weakly silicified *Pseudo-nitzschia* towards more heavily silicified *Fragilariopsis*. *Pseudo-nitzschia* remineralizes quickly in subsurface waters [65], while *Fragilariopsis* is a more efficient vector of carbon export [12]. Thus, enhanced primary production, in concert with potentially higher export efficiencies, could lead to a stronger downward flux of organic matter in Fe-replete areas under OA.

The described feedback by ‘CO_2_-fertilisation’, however, may not operate over the broad expanse of the Fe-limited SO. These regional differences in CO_2_-sensitivity might be even more pronounced in terms of carbon export efficiencies, as under Fe-depleted conditions no functional shifts in species composition were observed. Here, all assemblages were dominated by weakly silicified species such as *Pseudo-nitzschia* cf. *turgiduloides* [54] or *Synedropsis* sp. [60]. Frustules of both species are delicate and only preserved in shallow waters or under special circumstances such as large aggregation events in combination with anoxia [61], [65]. Irrespective of their potential for carbon export, all species dominating our incubations are ecologically important [61], [66], [67]. Furthermore, both *F. cylindrus* and *P. turgiduloides* are not only characteristic sea-ice algae but also dominate phytoplankton assemblages in open waters [66], [67]. In fact, most genera being characteristic for SO phytoplankton assemblages were present in initial and final phytoplankton assemblages (Table 3). Overall, species in the incubations resemble a mixture of typical open-ocean and sea-ice associated species [68]–[71]. Hence, our interpretations may not be restricted to sea-ice influenced habitats only.

Our results suggest that the potential ‘CO_2_ fertilization’ effect critically depends on the availability of Fe, determining how strongly the biological pump will serve as a carbon sink in the future SO. Realistic projections of primary production and CO_2_ sequestration thus remain difficult as long as scenarios for Fe input as well as its bioavailability to phytoplankton remain poorly constrained [18], [72]. The results of this study furthermore highlight the need to assess combined effects of important environmental factors in order to understand and predict responses to single stressors such as OA. In this respect, irradiance levels should also be considered as a potentially interacting factor. Indeed, the level of energization has been shown to strongly influence the strength of phytoplankton responses to OA [73], suggesting that also the interaction between Fe and CO_2_ availability could be modulated by light conditions. To thoroughly assess consequences of OA, multifactorial perturbation experiments (including factors such as different Fe sources or grazing) should target physiological as well as ecological responses of SO phytoplankton assemblages.
