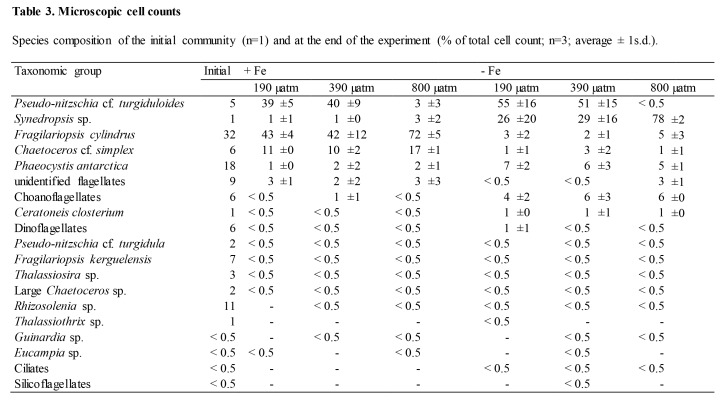# Correction: Iron Limitation Modulates Ocean Acidification Effects on Southern Ocean Phytoplankton Communities

**DOI:** 10.1371/annotation/c3c66438-6fc0-4b1a-ba1e-9ea637695e8b

**Published:** 2013-12-31

**Authors:** Clara J. M. Hoppe, Christel S. Hassler, Christopher D. Payne, Philippe D. Tortell, Björn Rost, Scarlett Trimborn

There was an error in the Author Contributions section. Only ST and CH should be noted as having conceived and designed the experiments.

There were formatting mistakes in Tables 1, 2, and 3 that affect the interpretation of the tables. The publisher apologizes for these mistakes. 

Please see the corrected Table 1 here: 

**Figure:**
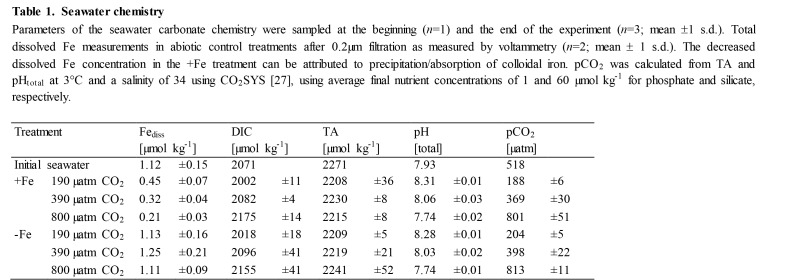


Please see the corrected Table 2 here: 

**Figure:**
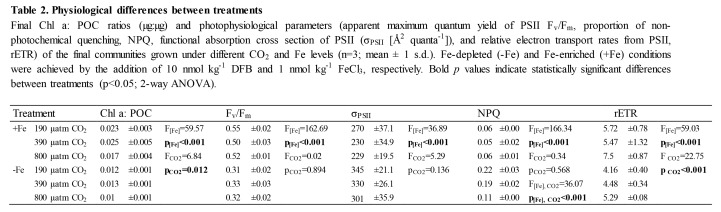


Please see the corrected Table 3 here: 

**Figure:**